# Macronutrient and micronutrient requirements in sickle cell disease across age groups: a narrative review

**DOI:** 10.1007/s12687-026-00959-8

**Published:** 2026-10-19

**Authors:** Sarbasree Bhattacharjee, Jarina Begum, Ranjitha S Shetty, Syed Irfan Ali, Gaurav Kumar Roy

**Affiliations:** 1 Department of Community Medicine, Manipal Tata Medical College, Manipal Academy of Higher Education, Manipal, India; 2 Department of Community Medicine, Kasturba Medical College, Manipal Academy of Higher Education, Manipal, India

**Keywords:** Sickle cell disease, Nutritional requirements, Macronutrients, Micronutrients, SCD

## Abstract

**Background:**

Sickle cell disease (SCD) is a hereditary haemoglobinopathy characterised by chronic haemolysis, inflammation, oxidative stress, and increased metabolic demands. Nutritional deficiencies worsen disease severity, growth, immunity, and quality of life, but evidence on age‑specific needs is fragmented. This narrative review synthesises current evidence on macronutrient and micronutrient requirements, deficiencies, and interventions in SCD across age groups.

**Methods:**

A structured literature search was conducted in PubMed, Scopus, and Google Scholar (January 2000-March 2025). Original studies evaluating nutritional status or interventions in confirmed SCD were included.

**Results:**

Individuals with SCD are in a hypermetabolic state with elevated energy and protein requirements. Common micronutrient deficiencies include vitamin D (prevalence 56.2% in children, 75.5% in adults), zinc, vitamin B12, folate, and vitamin C. Zinc deficiency is associated with more vaso‑occlusive crises, infections, delayed growth, and hospitalisations. Omega‑3 fatty acids, L‑arginine, glutamine, zinc, and vitamin D supplementation show potential benefits in reducing pain severity, vaso‑occlusive events, inflammation, and hospital admissions, while improving growth, endothelial function, and quality of life. However, the certainty of the evidence is low to very low due to small sample sizes and heterogeneous methods; adult‑specific data are particularly scarce.

**Conclusion:**

Major gaps remain in standardised dietary recommendations and supplementation protocols. Large, high‑quality randomised controlled trials are urgently needed to establish evidence‑based nutritional guidelines for SCD.

## Introduction

Sickle cell disease (SCD) is an inherited haemoglobin disorder characterised by polymerisation of sickle haemoglobin (HbS) under deoxygenated conditions, resulting in deformation of red blood cells from their normal biconcave shape to a characteristic sickle shape. SCD affects millions worldwide and is particularly prevalent among populations of African, Mediterranean, Middle Eastern, and South Asian ancestry.(Rees et al. 2010) SCD is caused by a homozygous or compound heterozygous mutation in the β-globin gene, resulting in the production of abnormal haemoglobin S (HbS). Individuals who inherit only one defective β-globin gene have sickle cell trait (SCT), which is usually asymptomatic but important for understanding carrier frequency and disease transmission. In contrast, SCD represents the homozygous or double-variant state that manifests clinically with recurrent pain crises, haemolytic anaemia, and multi-organ complications.(Inusa et al. 2019) India accounts for the largest number of people affected by SCD in South Asia, with more than 20 million individuals either living with the disease or carrying the trait. While SCT contributes to the genetic pool, regional disease risk, carrier frequency, and disease transmission, the present review focuses on individuals with SCD across different age groups.(Brousse and Rees 2021) The prevalence and health burden data presented here are therefore interpreted primarily in the context of SCD, with SCT details retained only for epidemiological background.(Hockham et al. 2018) Other inherited haemoglobin disorders, particularly thalassemia, also contribute substantially to the burden of haemoglobinopathies in these regions. However, thalassemia and other non-sickling haemoglobin disorders are beyond the scope of this review, which focuses on nutritional requirements and interventions in individuals with SCD.(Williams and Weatherall 2012) Although comprehensive data on the sickle cell trait (SCT) across Southeast Asia are limited, available mortality patterns provide critical insight into the underlying burden. Total deaths due to SCD were nearly 67 times higher than cause-specific deaths attributed to the condition in South Asia, highlighting substantial underdiagnosis and underreporting in the region. This discrepancy is particularly important in countries like India, where SCT prevalence is known to be high among the tribal community and historically malaria-endemic populations. Among children under the age of five globally, 81,100 deaths were attributed to SCD, making it the 12th leading cause of mortality in that age group. However, when deaths are attributed only to the underlying cause (per ICD standards), SCD drops to the 40th position, underscoring how traditional mortality reporting vastly underestimates the true impact of the disease.(Thomson et al. 2023) The GBD 2023 data estimate the burden of SCD and SCT at 0.059% and 0.22% of Disability-Adjusted Life Years, respectively, across India. Similarly, the estimates for Jharkhand put the burden of SCD and SCT at 0.057% and 0.26% of DALYs, respectively. In sheer numbers, the estimates are 7199.29 (5050.32-9771.61) and 32802.48 (19460.41-52052.16) for SCD and SCT, respectively.(Global Burden of Disease Collaborative Network 2024 HbS causes red blood cells to become stiff and sticky, leading to deformation into a sickle shape. These sickle-shaped cells flow poorly through small vessels, which can lead to many of the complications associated with SCA.(Bunn 1997) Clinical manifestations of SCD vary considerably among individuals. A major manifestation is recurrent severe pain due to vaso-occlusive crises, which occur when sickled red blood cells obstruct small vessels, contributing to ischaemia and tissue damage.(Kato et al. 2007) Nutrition plays a critical role in the health and clinical outcomes of adults living with SCD, with research highlighting the multifaceted impact of nutrient intake, deficiencies, and food environments on disease severity and quality of life.(Bell et al. 2024)SCD is associated with chronic inflammation, oxidative stress, and increased metabolic demands, collectively elevating energy and protein requirements compared with healthy individuals.(Hyacinth et al. 2010) Despite adequate dietary intake, a relative shortage of macro- and micronutrients has been documented, contributing to growth abnormalities, impaired immune function, and an increased risk of complications in this population. (dos Santos et al. 2018) Studies have shown that undernutrition can exacerbate clinical events and reduce patients’ independence, with significant deficiencies in essential micronutrients, including iron, zinc, vitamin D, calcium, and folic acid, frequently reported among individuals with SCD.(Bell et al. 2024) Interventions such as dietary supplementation with protein, amino acids, vitamins, and minerals have demonstrated potential benefits in improving growth and bone mineral density and in reducing the frequency of sickle-cell-related crises.(Hyacinth et al. 2010; dos Santos et al. 2018) Given the heightened metabolic state and the high prevalence of multiple nutrient deficiencies, understanding the full spectrum of essential macronutrients and micronutrients required by individuals of different age groups with SCD is a prerequisite for rational nutritional support. This narrative review synthesises current evidence on the types, functions, and required intakes of all major nutrients in individuals with SCD across age groups, with a focus on multiple interventions and geographic regions worldwide.

## Methods

### Study design

This article is a narrative (traditional) review of the literature on macronutrients and micronutrients in SCD. A narrative review was chosen over a systematic review or meta-analysis for the following reasons: the research question is broad, encompassing multiple nutrients, age groups, and clinical outcomes; the existing evidence is heterogeneous in study design and outcome measures; preliminary evidence suggests that quantitative synthesis (meta-analysis) would be limited by small sample sizes, variable definitions of deficiencies, and inconsistent supplementation protocols; the primary goal is to map the current landscape, identify evidence gaps, and provide a clinical overview rather than to produce a pooled effect estimate. (Collins and Fauser 2005; Green et al. 2006)

### Search strategy and selection criteria

A structured literature search was conducted to identify studies examining the role of nutrition in the management of Sickle Cell Disease (SCD). Searches were conducted in PubMed, Scopus, and Google Scholar from January 2000 to March 2025.

### Search terms and strategy

The following combinations of keywords and Boolean operators were used:

(“Sickle cell disease” OR “Sickle cell anaemia”) AND (“nutrition” OR “diet” OR “micronutrients” OR “macronutrients” OR “vitamins” OR “minerals”) AND (“management” OR “intervention” OR “dietary recommendations”).

### Literature search and selection

**Fig. 1:**
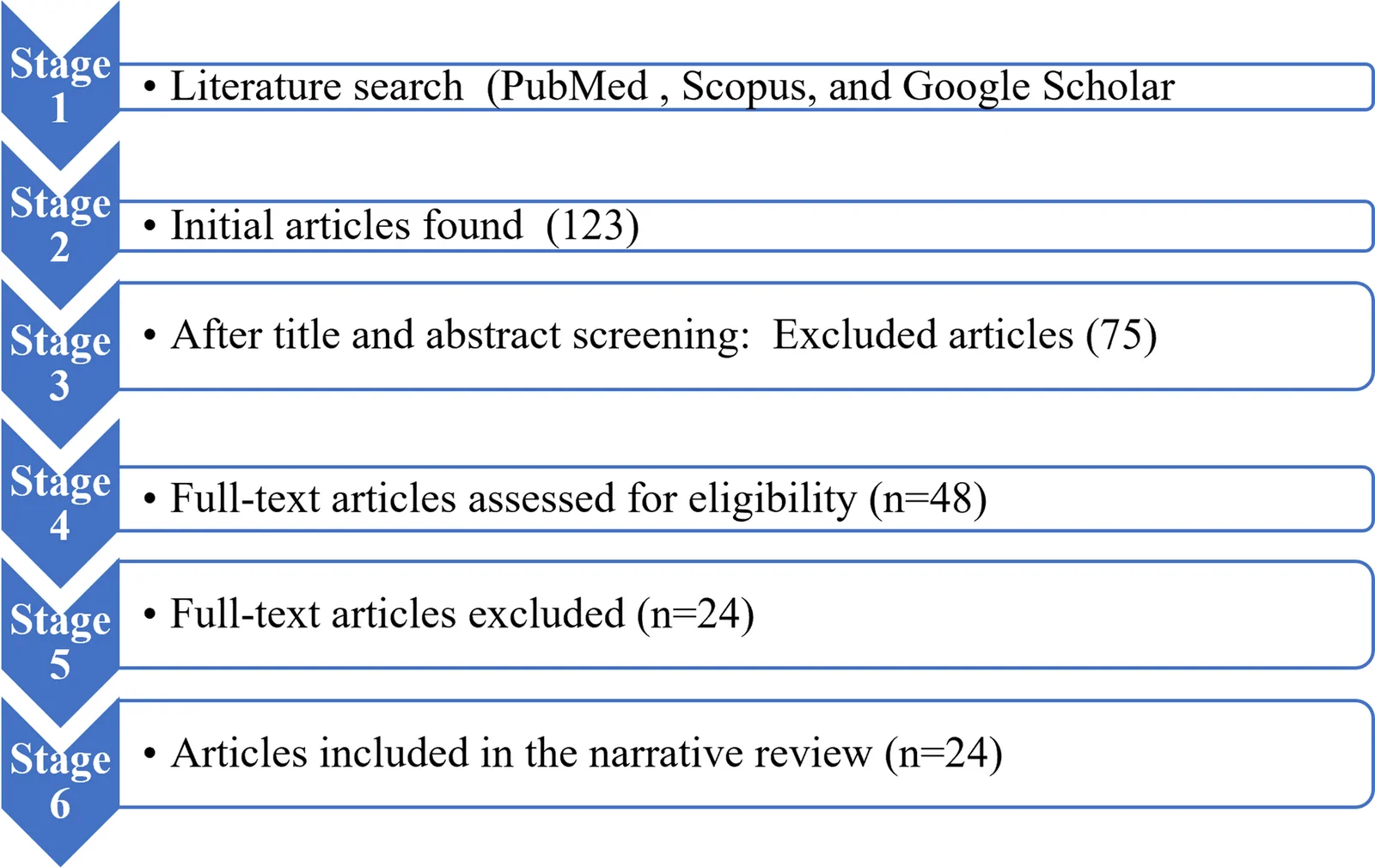
Study selection flow diagram showing the identification, title and abstract screening, full-text assessment, and final inclusion of articles in the narrative review

Electronic databases (PubMed, Google Scholar, Scopus) were searched for English-language articles combining terms for sickle cell disease and nutritional interventions (e.g., micronutrient supplementation, macronutrient modification, enteral nutrition, nutrition education). Reference lists of retrieved articles were also hand-searched. Studies were selected if they evaluated any nutritional intervention in individuals with confirmed SCD and reported clinical or patient-centred outcomes (vaso-occlusive crises, pain, hospitalisation, growth, quality of life, haematological or inflammatory markers linked to clinical change). Original research (randomised controlled trials, pre-post studies, cohort, and case-control studies) formed the core evidence. Purely observational dietary surveys without intervention, animal or in vitro experiments, studies not available in full text, and non-peer-reviewed sources were excluded. Selection was purposive and iterative, guided by the central review question; greater interpretive weight was placed on studies with comparator groups and direct clinical endpoints. The study selection process is summarised in Fig. 1.

### Review process

Two independent reviewers (SB and JB) screened titles and abstracts, followed by full-text evaluation. Any discrepancies were resolved through discussion and consensus with the other authors (RS, GR, and SIA). The manuscript was drafted, critically reviewed, and approved by all authors to ensure accuracy, coherence, and methodological transparency. To minimise the risk of double-counting, data extraction and synthesis prioritised primary studies. Review articles were used to support contextual understanding and to identify additional eligible studies but were not included as an independent source of evidence in the synthesis. A detailed quality appraisal of each included study, including the assessment tool used and key limitations, is provided in Table 1.

### Evidence synthesis and interpretation

Given the heterogeneity in study designs, populations, interventions, and outcomes, evidence was synthesised narratively and organised by nutrient and, where available, by age group. Interpretation considered study design, methodological quality, sample size, risk of bias, and consistency of findings. Greater interpretive weight was given to methodologically stronger evidence, while findings from smaller or observational studies were interpreted cautiously. Where findings were inconsistent, they were reported as such, with differences considered in relation to study populations, designs, interventions, and methodological limitations. Quality appraisal informed interpretation but was not used as an exclusion criterion. No quantitative pooling was undertaken.

**Table 1:** Quality appraisal of each included study

Serial No.	Article	Study Type	Appraisal Tool	Quality Rating	Key Limitations
1	The Role of Nutrition in Sickle Cell Disease	Narrative Review	SANRA	Moderate	No systematic search strategy; selective citation; no quality assessment of primary studies
2	Nutrition in sickle cell disease: recent insights	Narrative Review	SANRA	Moderate	No formal quality appraisal; some recommendations lack direct citations (e.g., hydration
3	Antioxidant supplementation for sickle cell disease	Cochrane systematic review with meta‑analysis	AMSTAR‑2 / GRADE	High	Some outcomes were downgraded to low/very low certainty due to imprecision and indirectness
4	Effect of omega-3 (n-3) fatty acid supplementation in patients with sickle cell anemia: Randomised, double-blind, placebo-controlled trial	Randomised controlled trial (double‑blind)	Cochrane RoB 2	Low risk of bias	No prospective registration was mentioned
5	A Meta-analysis on Vitamin D Deficiency in Patients with Sickle Cell Disease	Meta‑analysis of cross‑sectional studies	AMSTAR‑2	Moderate	High heterogeneity (I² > 90%); publication bias not fully assessed
6	Prevalence of Vitamin D deficiency in sickle cell disease: A systematic review	Systematic review (no meta‑analysis)	AMSTAR‑2	Moderate	No meta‑analysis; heterogeneity not quantified; risk of bias of primary studies not formally assessed
7	Vitamin D Supplementation Improves Health-Related Quality of Life and Physical Performance in Children with Sickle Cell Disease and in Healthy Children	Randomised controlled trial (double‑blind)	Cochrane RoB 2	Low risk of bias	Very small sample (*n* = 21); comparisons between vitamin D doses (no placebo control)
8	Vitamin D levels are low in adult patients with sickle cell disease in Jamaica and West Africa	Cross‑sectional (multi‑centre)	JBI checklist	Moderate	Convenience sampling; no blinding of lab analysts; small Jamaica sample (*n* = 20)
9	Sickle cell bone disease: Response to vitamin D and calcium	Pre‑post intervention (no control group)	JBI for quasi‑experimental	Low	No control group; small sample (*n* = 14); attrition not reported; no blinding
10	Vitamin B supplementation for sickle cell disease	Cochrane protocol (no results)	N/A	N/A	Protocol only; no empirical data; not suitable for outcome synthesis
11	Vitamin B12 deficiency in sickle cell patients at the Bamako sickle cell research and control center: About two cases	Case series (*n* = 2)	JBI for case reports	Very low	Only two cases; no generalisability; descriptive only
12	Zinc in sickle cell disease: A narrative review	Narrative review	SANRA	Moderate	No systematic search; explicitly notes lack of adult data (a valid gap statement)
13	Plasma Zinc and Magnesium Levels in Sickle Cell Disease Patients in Latakia, Syria	Cross‑sectional	JBI checklist	Moderate	Small sample (*n* = 64 SCD, 32 controls); single centre; convenience sampling
14	Serum Zinc Level during and after Acute Painful Episodes in Children with Sickle Cell Anemia at the Aminu Kano Teaching Hospital, Kano, Northern Nigeria	Cross‑sectional (within‑subject comparison)	JBI checklist	Moderate	Small sample (*n* = 45); no blinding; single time point per episode
15	Effect of zinc supplementation on growth and body composition in children with sickle cell disease	Randomised controlled trial (double‑blind)	Cochrane RoB 2	Low risk of bias	Older trial (2002); sample size *n* = 38; still methodologically sound
16	Zinc supplementation decreases oxidative stress, incidence of infection, and generation of inflammatory cytokines in sickle cell disease patients	Randomised controlled trial (double‑blind)	Cochrane RoB 2	Low risk of bias	Small sample (*n* = 36); short duration (3 months)
17	Red Blood Cell Vitamin C Concentration and Its Effect on Deformability in Pediatric Sickle Cell Disease	Cross‑sectional (laboratory‑based)	JBI checklist	Moderate unclear	Very small sample (*n* = 20 SCD, 12 controls); no adjustment for dietary confounders
18	Effects of Vitamin C Supplementation on Sickle Cell Disease	Quasi‑experimental (pre‑post, no control)	JBI for quasi‑experimental	Low	No control group; high attrition; blinding; journal indexing concerns
19	Assessing and managing iron deficiency anemia in sickle cell disease: Insights from a systematic review and meta-analysis	Systematic review & meta-analysis	AMSTAR-2	Moderate	Only two databases; all primary studies cross‑sectional (no RCTs); high heterogeneity (I²=88.8%); publication bias detected; variability in IDA diagnostic criteria
20	Iron restriction in sickle cell disease: When less is more	Narrative review	SANRA	Moderate	No systematic search strategy (MEDLINE search described but not systematic); no formal quality assessment of primary studies; article funded by CSL Vifor (manufacturer of vamifeport); explicit statement: “no formal grading of the quality of evidence was performed”
21	Micronutrient levels and haemato-biochemical status of patients with sickle cell anaemia at a tertiary hospital in Abakaliki, south-eastern Nigeria: a cross-sectional study	Cross-sectional	JBI Checklist for Cross‑Sectional Studies	Moderate	Convenience sampling (consecutive enrolment at single centre); no blinding of laboratory analysts; inability to assess intracellular micronutrient levels due to financial constraints; no quantification of dietary intake
22	Mineral bone disorders and kidney disease in hospitalised children with sickle cell anemia	Prospective cross‑sectional	JBI Checklist for Cross‑Sectional Studies	Moderate	Single centre (Uganda); no bone density or biopsy assessments; underpowered for mortality analysis (only 6 deaths); unable to differentiate acute kidney disease from chronic kidney disease
23	Zinc, Magnesium, and Copper Levels in Patients with Sickle Cell Disease: A Systematic Review and Meta-analysis	Systematic review & meta‑analysis	AMSTAR‑2	Moderate	Protocol not registered in PROSPERO (authors state as limitation); only English‑language studies included; substantial heterogeneity (I² 92–97%); publication bias detected for zinc (Egger’s *p* = 0.042); limited generalisability due to lack of clinical outcome data
24	Magnesium for treating sickle cell disease	Systematic review & meta‑analysis	Cochrane RoB	Moderate	Evidence downgraded to moderate/low due to risk of bias (unclear blinding of outcome assessors, incomplete outcome data in one study), imprecision (wide CIs), and heterogeneity; one oral Mg study terminated early with small sample (*n* = 12); most outcomes could not be meta‑analysed due to insufficient data

## Results

The following synthesis is based exclusively on open-access, peer-reviewed literature indexed in PubMed, Scopus and Google Scholar. A total of **24 articles met** the inclusion criteria and were included in the narrative synthesis. Findings were synthesised according to nutrient category and, where age-specific data were available, by age group. Studies reporting mixed or unspecified age groups were presented according to the populations reported in the original studies.

### Macronutrients

#### Energy (carbohydrate and fat) and protein requirements

##### Children (aged 3–6 years)

A study of 41 children with SCD and 31 healthy controls, aged 3–6 years, measured dietary intake and resting metabolic rate (RMR). Children with SCD had significantly elevated RMR compared with healthy controls after adjusting for gender and weight. Energy intake was similar between groups, but the energy intake-to-RMR ratio was significantly lower in the SCD group, indicating a relative energy deficiency. It was concluded that pre-pubertal children with SCD require increased energy from macronutrients (proteins, carbohydrates, and fats) and that undernutrition is a primary clinical manifestation of this relative nutrient deficiency.(Umeakunne and Hibbert 2019).

##### Adolescents

No separate adolescent-only studies establishing energy or protein requirements were identified in the included literature.(Umeakunne and Hibbert 2019) Therefore, specific energy and protein requirements for adolescents with SCD could not be determined from the available evidence.

##### Adults

Studies in adults with SCD have reported increased resting energy expenditure and protein turnover compared with healthy controls, suggesting increased dietary energy and protein requirements. However, specific SCD-related dietary requirements or recommended dietary allowances for adults have not been established.(Umeakunne and Hibbert 2019).

#### Amino acids: arginine

##### Children and adolescents

A randomised, double-blind, placebo-controlled trial in children with SCD hospitalised for vaso-occlusive pain (n = 68, aged 5–17 years) found that oral L-arginine hydrochloride (100 mg/kg three times daily) reduced pain severity compared with placebo. The mean change in pain scores (0–10 visual analogue scale) was − 1.41 (95% CI -1.65 to -1.18), favouring arginine.(Bolarinwa et al. 2024) Another study in children (n = 28, aged 3–29 years) showed that arginine reduced opioid consumption (MD -1.77 mg/kg, 95% CI -2.97 to -0.57). The evidence for reducing hospital days in children was very uncertain (MD -0.85 days, 95% CI -1.87 to 0.17; very low certainty).(Bolarinwa et al. 2024).

##### Adults

A study of 20 adults with SCA and 20 healthy controls receiving L-arginine (1 g/day for 6 weeks) found that baseline plasma arginine and nitric oxide metabolite levels were significantly lower in participants with SCA (p < 0.001 and p < 0.05, respectively).(Umeakunne and Hibbert 2019) Following supplementation, the percentage increases in plasma arginine and nitric oxide metabolites were significantly greater in participants with SCA than in controls (p < 0.001 for both).(Umeakunne and Hibbert 2019) The Cochrane review included two adult studies (125 participants) and concluded that L-arginine may reduce pain severity but may not reduce crisis frequency or haemoglobin levels.(Bolarinwa et al. 2024).

#### Amino acids: glutamine

##### Children and adolescents

In a study of 27 children and adolescents with SCD receiving oral glutamine (600 mg/kg/day) for 24 weeks, median resting energy expenditure decreased by 6% using the Harris-Benedict equation (p = 0.053) and by 5% using a modified equation (p = 0.049). Improvements in nutritional parameters were also reported.(Umeakunne and Hibbert 2019).

##### Adults

In 2017, the US Food and Drug Administration approved pharmaceutical-grade L-glutamine for children and adults with SCD aged ≥ 5 years who have recurrent vaso-occlusive pain events. The recommended dose is 0.3 g/kg twice daily, with a maximum daily dose of 30 g. No adult-specific efficacy trial details were provided in the reviewed open-access articles beyond the approval statement.(Umeakunne and Hibbert 2019).

#### Omega-3 fatty acids

##### Children, adolescents, and adults

A randomised, double-blind, placebo-controlled trial in Sudan enrolled 140 patients with homozygous SCD aged 2–24 years. Daily omega-3 capsules (each containing 277.8 mg DHA and 39.0 mg EPA) were given according to age and weight: 1 capsule for ages 2–4 years (median weight 13 kg), 2 capsules for ages 5–10 years (median weight 25 kg), 3 capsules for ages 11–16 years (median weight 37 kg), and 4 capsules for ages ≥ 17 years (median weight 51 kg).(Daak et al. 2013) After one year of treatment, the omega-3 group had a median rate of clinical vaso-occlusive events of 0 per year compared with 1.0 per year in the placebo group (p < 0.0001). The incidence of severe anaemia (haemoglobin < 50 g/L) was 3.2% in the omega-3 group vs. 16.4% in the placebo group (p < 0.05). Blood transfusion rates were 4.5% vs. 16.4% (p < 0.05). White blood cell count decreased significantly in the omega-3 group (from 15.6 ± 4.0 to 14.4 ± 3.3 × 10³/µL, p < 0.05). School attendance improved: the odds of being unable to attend school at least once due to SCD-related illness were reduced to 0.4 (95% CI 0.2–0.9, p < 0.05). The trial reported reductions in vaso-occlusive events, severe anaemia, and transfusion rates, as well as improved school attendance.(Daak et al. 2013) However, a Cochrane review rated the evidence for most omega-3 outcomes as very low certainty, reflecting the limited evidence base. (Bolarinwa et al. 2024)

### Micronutrients

#### Iron

##### Children, adolescents and adults

For individuals with SCD, interpreting iron status is more complex than in the general population because chronic haemolysis, which releases iron, is counterbalanced by increased erythropoietic demand, which may deplete iron stores. The diagnostic value of serum ferritin is limited in SCD because chronic inflammation commonly elevates ferritin levels, masking true iron deficiency. A systematic review and meta-analysis of 14 studies covering 763 SCD cases with 118 instances of iron deficiency anemia (IDA) found a pooled IDA prevalence of 6% (95% CI: 1%-20%) among individuals with SCD, with high heterogeneity (I² = 88.8%); sensitivity analysis adjusted for publication bias indicated an 8% prevalence (95% CI: 4%-19%) with reduced heterogeneity (I² = 19.5%).

The review further highlighted the potential adverse clinical consequences of untreated IDA in SCD and emphasised the importance of early diagnosis and targeted management.(Jose et al. 2025) Conversely, a critical review examined the hypothesis that iron restriction may have therapeutic potential in SCD. The proposed rationale is that reducing erythrocyte haemoglobin concentration may lower intracellular HbS concentration, thereby reducing HbS polymerisation. However, the review emphasised the need for well-designed clinical studies to determine whether iron restriction is a safe and effective therapeutic strategy in SCD.(Castro et al. 2024) A cross-sectional study of 60 participants with SCA and 60 HbAA controls in south-eastern Nigeria found that plasma iron levels were significantly higher in the SCA group compared with controls. Iron overload may occur in SCA due to multiple blood transfusions and chronic haemolysis, and caution should be exercised when considering iron supplementation in the absence of confirmed iron deficiency.(Nnachi et al. 2022).

#### Calcium

##### Children and young adults

Calcium status in SCD is closely intertwined with vitamin D metabolism and bone health. A 12-month clinical trial in 14 adults with SCD evaluated the effects of vitamin D₂ (50,000 IU weekly for 8 weeks, then every other week for 44 weeks) plus calcium carbonate (1,000 mg/day).(Adewoye et al. 2008) Hypocalcaemia was observed in 14% of patients, with serum calcium levels significantly lower in individuals with SCD compared with controls, and 12% of patients had 25(OH)D concentrations below the normal range. The study reported a hypercalcaemic tendency associated with elevated PTH levels in SCD; the authors suggested that impaired intestinal absorption of calcium and vitamin D may contribute to disturbed calcium metabolism and skeletal changes.(Batte et al. 2023).

#### Magnesium

##### Children, adolescents and adults

Magnesium status may be compromised in individuals with SCD, and supplementation has been investigated for its potential role in preventing sickle red blood cell dehydration. A systematic review and meta-analysis of 36 studies evaluated serum levels of zinc, magnesium, and copper in individuals with SCD. The pooled analysis found that individuals with SCD had significantly lower magnesium levels compared with healthy controls (standardised mean difference [SMD] = -0.53 [95% CI: -1.0 to -0.06], p = 0.026). SCD patients may have lower magnesium levels than those without the disease.(Elkhidir et al. 2022) A cross-sectional study in south-eastern Nigeria reported significantly lower plasma magnesium levels in adults with SCA than in HbAA controls (9.65 ± 1.38 vs. 19.38 ± 6.37 mg/dL; p < 0.001).(Nnachi et al. 2022) A Cochrane review included five randomised placebo-controlled studies involving 386 participants aged 3–53 years. Two studies evaluated intravenous magnesium sulphate, and three evaluated oral magnesium pidolate. The review found no clear benefit of intravenous magnesium for pain, length of hospital stays, or health-related quality of life, while oral magnesium did not reduce the frequency of painful episodes. The authors concluded that the available evidence was insufficient to establish a clinical benefit of intravenous or oral magnesium therapy in SCD. (Than et al. 2017)

#### Zinc

##### Children and adolescents

A narrative review reported that zinc insufficiency/deficiency occurs in up to 57% of children with SCD.(Miranda et al. 2022) There is a direct association between low zinc levels and increased disease severity, including more frequent vaso-occlusive crises, hospitalisations, growth retardation, and delayed wound healing.(Miranda et al. 2022) A study from Syria (children and adolescents) found that 14.1% of individuals with SCDwere zinc-deficient. (Yousef et al. 2025) In Nigerian children with SCA, serum zinc levels were significantly lower during acute painful crises than after resolution, suggesting increased demand and utilisation during crises.(Kudirat et al. 2019) Zinc supplementation (10 mg/day elemental zinc) positively affected growth and body composition in children with SCD.(Zemel et al. 2002) It also reduced the incidence of infections and hospital admissions.(Bao et al. 2008).

##### Adults

The narrative review explicitly states: “Unfortunately, adults don’t have the same follow-up, and there is only little/non-satisfying data available in the literature as far as we can see. New research, especially for adults, is needed to improve survival rates in this population.(Miranda et al. 2022) One adult study showed that zinc supplementation (25 mg three times daily for 3 months) reduced infections and hospital admissions, but no other adult‑specific prevalence or outcome data were reported.(Bao et al. 2008; Miranda et al. 2022) The Cochrane review included one study of 36 participants (age not specified) and found that zinc may not reduce the frequency of crisis (rate ratio 0.62, 95% CI 0.17–2.29; low‑certainty evidence) but may increase haemoglobin level (MD 1.26 g/dL, 95% CI 0.44–1.26; low‑certainty evidence).(Bolarinwa et al. 2024) The evidence for complete healing of leg ulcers was very uncertain (RR 2.00, 95% CI 0.60–6.72; 1 study, 34 participants; very low‑certainty evidence).(Bolarinwa et al. 2024).

#### Vitamin D

##### Children

A meta-analysis of 14 studies including children with SCD reported a pooled prevalence of vitamin D deficiency (25(OH)D < 20 ng/mL) of 56.2% (95% CI 36.7–75.7*)*.(Mohamed et al. 2020) Severe deficiency (< 10 ng/mL) occurred in 27.0% of all individuals with SCD, children and adults combined. A systematic review found that prevalence estimates ranged from 56.4% to 96.4% in SCD populations, depending on the definitions used.(Nolan et al. 2015) A randomised trial in children with HbSS (*n* = 21, aged 5–20 years) receiving daily high-dose vitamin D₃ (4,000 or 7,000 IU) for 12 weeks showed significant improvements in Patient‑Reported Outcomes Measurement Information System (PROMIS) t‑scores for pain (reduction), fatigue (reduction), depressive symptoms (reduction), and upper‑extremity function (increase). (Dougherty et al. 2020) Physical performance also improved: peak power (watts) and isometric maximal voluntary contraction torque at dorsiflexion increased significantly. The total Bruininks‑Oseretsky Test of Motor Proficiency score improved in both children with HbSS and healthy controls. (Dougherty et al. 2020)

##### Adults

The meta-analysis of 7 adult studies found a much higher pooled prevalence of vitamin D deficiency: 75.5% (95% CI, 57.5–93.5). This difference between children and adults was statistically significant (χ² = 28.3, p < 0.001).(Mohamed et al. 2020) In a study of adult individuals with SCD from Jamaica (n = 20) and West Africa (n = 50), mean 25(OH)D levels were 37% and 39% lower than in population controls, respectively (Jamaica: 45.4 ± 16.7 vs. 72.2 ± 17.8 nmol/L; West Africa: 46.5 ± 12.8 vs. 75.9 ± 17.2 nmol/L; p < 0.05).(Tayo et al. 2014) The authors suggested that metabolic demands on the liver in SCD may reduce the capacity to synthesise vitamin D‑binding protein.(Tayo et al. 2014) A 12‑month supplementation study in 14 adults with SCD (11 HbSS, 3 HbSC; mean age 28.1 ± 6.7 years) using oral vitamin D₂ (50,000 IU weekly for 8 weeks, then every other week for 44 weeks) plus calcium carbonate (1,000 mg/day) normalised 25(OH)D levels from 11.6 ± 4 to 34.6 ± 11 ng/mL (p < 0.001).(Adewoye et al. 2008) Bone mineral density increased significantly at the lumbar spine (+ 3.6%, p = 0.009) and femoral neck (+ 4.6%, p = 0.05). Markers of bone resorption (CTx) and formation (osteocalcin) remained unchanged, suggesting that increased bone resorption may persist despite vitamin D repletion. (Adewoye et al. 2008)

#### Vitamin B complex

##### Children and adults

A Cochrane review protocol for vitamin B supplementation in SCD was published in 2022, but results are not yet available. The protocol states that daily folic acid supplementation (1 mg/day) is common in SCD due to presumed folate deficiency in the setting of haemolytic anaemia and rapid red blood cell turnover. Although folic acid supplementation may increase serum folate levels, its effects on anaemia and related symptoms remain unclear.(Francis et al. 2022).

#### Vitamin B12

##### Adults

Two case reports from the Bamako Sickle Cell Research Centre described women with SCD who developed acute anaemia due to vitamin B12 deficiency. The deficiency was difficult to diagnose because symptoms overlap with SCD itself, and folic acid supplementation can mask haematological abnormalities while not preventing neurological damage. The mechanism involves increased demand, inadequate supply, and reduced absorption due to sickling of gastric blood vessels, which reduces intrinsic factor production.(Kanta et al. 2023).

#### Vitamin B6

##### All ages

Vitamin B6 deficiency has been identified in patients with sickle cell anaemia, with low circulating levels observed and some clinical improvement following supplementation. Low levels of B6 and relative folate deficiency are associated with hyperhomocysteinaemia, a risk factor for cardiovascular disease and stroke in SCD.(Hyacinth et al. 2010).

#### Vitamin C and other antioxidants

##### Children

Vitamin C concentrations are significantly lower in paediatric individuals with SCD than in healthy controls, potentially due to increased reactive oxygen species that deplete vitamin C stores.(Choi et al. 2023) Supplementation may improve certain blood parameters, particularly in patients with the SC genotype, but longer-term studies are needed.(Adiiboka et al. 2019).

##### Adults and children

The Cochrane review assessed high-dose vitamin C (1400 mg) plus vitamin E (800 mg) versus placebo in one study of 83 participants (adults).(Bolarinwa et al. 2024) The evidence was rated as having very low certainty. The intervention did not reduce the frequency of crisis (RR 1.18, 95% CI 0.64–2.18) or pain severity (RR 1.33, 95% CI 0.40–4.37). There was a possible increase in acute chest syndrome (RR 2.66, 95% CI 0.77–9.13). Adverse effects (headache, nausea, fatigue, diarrhoea, epigastric pain) were similar between groups (RR 0.56, 95% CI 0.31-1.00). Haemoglobin levels (median [IQR]) were 90 g/L (81–96) in the vitamin group vs. 93.5 g/L (84–105) in the placebo group.(Bolarinwa et al. 2024).

N-acetylcysteine (NAC;1200 mg) versus placebo, one study of 96 participants (age ≥ 12 years, including adolescents and adults) found that NAC may not reduce crisis frequency (rate ratio 0.99, 95% CI 0.53–1.84), pain severity (MD 0.17, 95% CI -0.53 to 0.87), or haemoglobin level (MD-0.18 g/dL, 95% CI -0.40 to 0.04). All evidence was of low or very low certainty. For L‑arginine, as detailed earlier, the Cochrane review found low‑certainty evidence for pain reduction, but no benefit for crisis frequency or haemoglobin.(Bolarinwa et al. 2024).

### Water and hydration

#### All ages

Adequate water intake and hydration may help limit erythrocyte dehydration and the increased blood viscosity. Poorly hydrated erythrocytes become more viscous, contributing to vaso-occlusive crises. Even those with sickle cell trait can experience blood viscosity during strenuous sports. Recommendations include drinking 8–10 glasses of water daily, limiting dietary sodium intake, avoiding excessive sweating, and drinking fluids frequently throughout the day. No age-specific differences in hydration requirements were reported in the reviewed articles.(Umeakunne and Hibbert 2019) Fig. 2 summarises the key macronutrient and micronutrient imbalances commonly observed in SCD and their downstream clinical consequences.

**Fig. 2:**
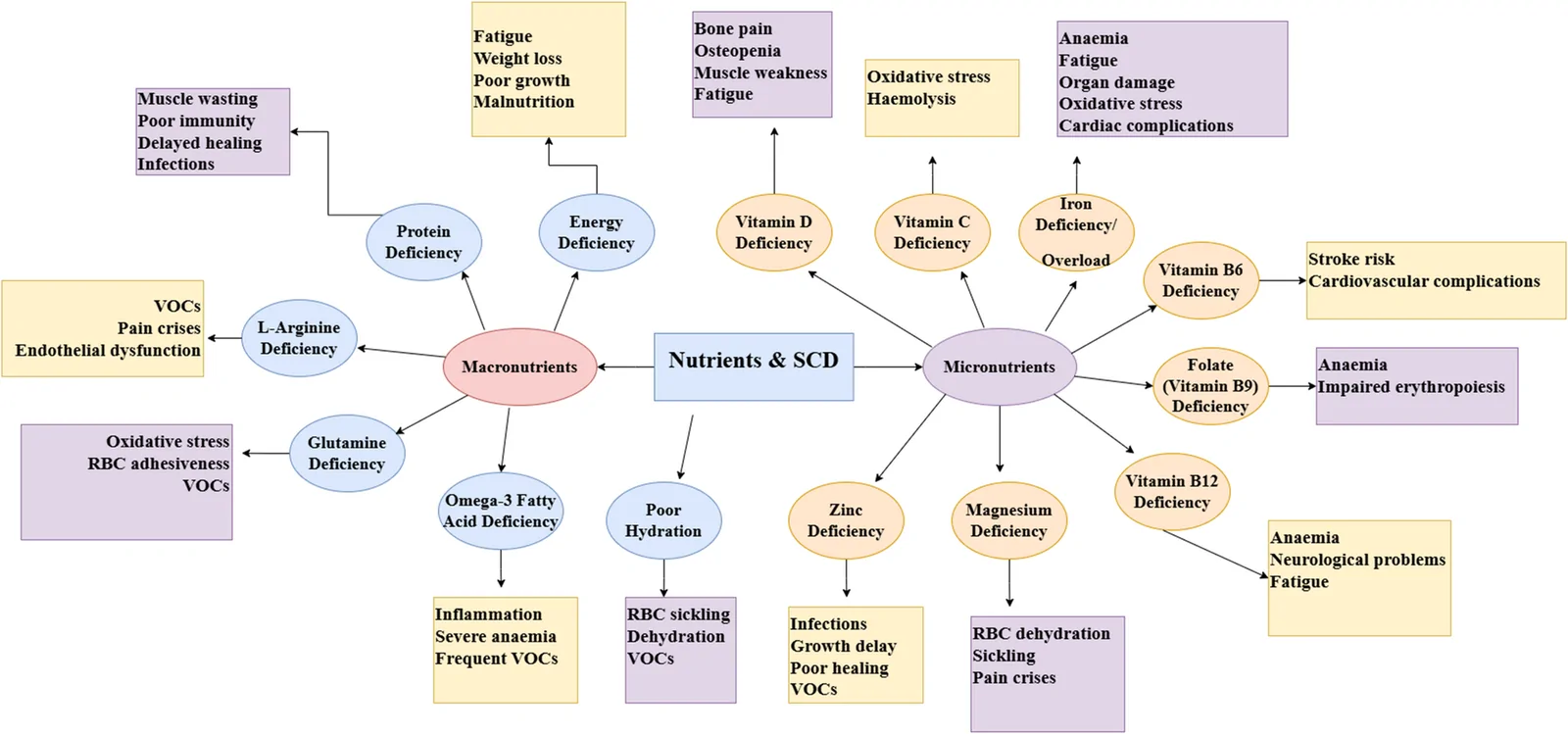
Macronutrient and micronutrient imbalances commonly observed in SCD and their downstream clinical consequences

## Discussion

The strength of evidence varied across nutrients and study designs. Systematic reviews and randomised controlled trials were given greater interpretive weight when assessing the effects of supplementation, whereas observational studies were primarily considered for evidence on nutritional status, deficiencies, and associations with clinical outcomes. Findings from smaller uncontrolled studies and narrative reviews were interpreted as supportive, where stronger evidence was unavailable. Importantly, evidence of nutrient deficiency was distinguished from evidence demonstrating clinical benefit from supplementation.

### General landscape of nutritional research in SCD

The overall body of literature on nutrition in SCD is dominated by descriptive studies with limited intervention rigour. A systematic review of nutritional research in sub-Saharan Africa found that most investigations were descriptive, with only a small proportion interventional. (Nartey et al. 2021) A recent systematic review and meta-analysis of nutritional supplementation for children and adolescents with SCD included 20 randomised controlled trials (*n* = 2058) analysing 9 dietary supplements and concluded that the evidence for many nutrients, including vitamin A, magnesium sulphate, and zinc, remained limited and of poor quality. (Orsi et al. 2025) A narrative review on nutrition in SCD similarly highlighted the paucity of large trials supporting the benefits of nutritional supplementation.(Ohemeng and Boadu 2018) A 2024 Cochrane review on antioxidant supplementation concluded that the evidence supporting the effect of the interventions was of very low to low certainty for the review’s primary outcomes, and the evidence for the secondary outcomes was inconclusive.(Bolarinwa et al. 2024) A recent scoping review on traditional medicine in SCD noted that several medicinal plants demonstrated anti-sickling, antioxidant, anti-inflammatory, and immunomodulatory properties, and formulations such as Niprisan^®^, Ciklavit^®^, and T AYU HM™ Premium showed potential in reducing vaso-occlusive crises and improving haematological parameters, further indicating that many potential therapeutic substances in SCD remain understudied.(Javed et al. 2026) The type of evidence and its overall interpretation across nutritional factors and interventions are summarised in Table 2.

**Table 2:** Synthesis of evidence for nutritional factors and interventions in sickle cell disease

Nutritional factor	Evidence represented	Overall evidence	Interpretation
Macronutrients (energy, protein, carbohydrate and fat)	Metabolic, observational, and limited intervention evidence	Increased energy expenditure and altered protein metabolism have been reported; evidence for SCD-specific carbohydrate and fat requirements is limited	Increased energy and protein needs are suggested, but precise age-specific requirements and SCD-specific macronutrient intake targets remain insufficiently established
L-arginine	RCTs and systematic review evidence	Some trials report improvement in pain-related outcomes; evidence is low certainty for some outcomes	Potential benefit, but insufficient evidence for routine use
Glutamine	Intervention evidence and review evidence	Potential metabolic and clinical benefits reported	Evidence is limited; age-specific nutritional requirements remain unclear.
Omega-3 fatty acids (DHA/EPA)	RCTs, observational studies and systematic reviews	Individual studies report favourable outcomes; systematic review evidence is of very low certainty for most outcomes	Individual trial findings are favourable, but overall evidence remains uncertain.
Iron	Observational studies, systematic review/meta-analysis and narrative review	Iron deficiency occurs in some patients, whereas iron excess may occur depending on transfusion and clinical status	Iron status should be individually assessed; routine supplementation cannot be generalised
Calcium	Observational and small intervention evidence	Altered calcium status and bone-health abnormalities reported, often alongside vitamin D abnormalities	Evidence is insufficient to establish SCD-specific calcium supplementation requirements.
Magnesium	Observational/meta-analytic and randomised evidence	Lower magnesium levels reported; randomised evidence shows no clear clinical benefit from supplementation	Low magnesium status should not be equated with evidence for routine supplementation
Zinc	Observational studies, small RCTs/interventions and review evidence	Low zinc status and adverse clinical associations reported; intervention studies suggest possible benefits	Potential benefit, particularly in deficiency, but insufficient evidence for universal supplementation
Copper	Primarily observational/meta-analytic evidence	Pooled copper levels were higher in SCD than in controls.	Clinical implications and supplementation requirements remain insufficiently established
Vitamin D	Observational studies, systematic reviews/meta-analyses and intervention studies	Deficiency is frequently reported; supplementation findings for clinical outcomes remain heterogeneous	Evidence for deficiency is stronger than evidence for SCD-specific clinical benefit from supplementation
Folate/folic acid (vitamin B9)	Observational/review evidence	Supplementation is commonly used, but effects on anaemia and clinical outcomes remain uncertain	Evidence is insufficient to establish uniform SCD-specific supplementation requirements
Vitamin B12	Case reports evidence	Deficiency has been reported and may be difficult to distinguish clinically from manifestations of SCD	Evidence supports awareness and assessment where clinically indicated; intervention evidence is limited
Vitamin B6	Limited observational/intervention evidence	Low levels have been reported and linked with hyperhomocysteinaemia	Evidence is limited; no firm SCD-specific supplementation recommendation can be made
Vitamin C	Observational and limited intervention evidence	Lower concentrations reported in children; limited supplementation studies report selected changes	Evidence is insufficient to establish routine supplementation
Vitamin E	Limited randomised/systematic-review evidence, mainly in combination with vitamin C	High-dose vitamin C plus vitamin E has not demonstrated clear clinical benefit; evidence is very low certainty	No established clinical benefit of the evaluated vitamin C plus vitamin E combination.
N-acetylcysteine (NAC)	Randomised/systematic-review evidence	No clear benefit for crisis frequency, pain severity or haemoglobin; evidence low/very low certainty	Current evidence does not establish clinical benefit
Water and Hydration	Narrative/review evidence	Hydration is discussed as relevant to erythrocyte dehydration and viscosity; age-specific requirements were not identified	General hydration is relevant, but evidence does not establish SCD-specific age-based fluid requirements

### Macronutrient requirements: energy, protein and hydration

A consistent finding across multiple studies is that individuals with SCD are in a hypermetabolic state with elevated energy and protein requirements. A recent analysis of the National Inpatient Sample (2016–2020) found protein-energy malnutrition in 25.9% of individuals with SCD. Protein-energy malnutrition was associated with more than two-fold higher odds of mortality, longer hospital stays, and higher hospital costs.(Ramesh et al. 2025) Erythrocytes dehydration increases viscosity and may contribute to the vaso-occlusive events; frequent fluid intake of water and limitation of high-sodium processed foods have therefore been recommended. (Umeakunne and Hibbert 2019)

### Micronutrient deficiencies

Vitamin D deficiency is highly prevalent across all age groups with SCD.(Mohamed et al. 2020) It is associated with impaired bone metabolism in children.(Mulyana et al. 2024) Vitamin D3 may reduce respiratory complications and shorten hospital stays, though further studies are needed.(da Conceicao Souza et al. 2025) Vitamin D deficiency was prevalent in 80% of those with B12 deficiencies and 90.4% of those with normal B12 levels, highlighting the co-occurrence of multiple micronutrient deficiencies.(Ononogbu et al. 2024).

Vitamin D supplementation probably increased serum 25(OH)D levels and reduced pain days compared with placebo at 8 weeks.(Soe et al. 2020) Overall, observational and pooled evidence indicate a substantial burden of vitamin D deficiency in SCD; however, evidence that supplementation improves SCD-specific clinical outcomes remains limited and heterogeneous. A large cohort study of individuals with SCD in Tanzania across all age groups found severely low vitamin B12 levels.(Kisali et al. 2024) A 2024 cross-sectional study reported a 27% prevalence of B12 deficiency in adults with SCD and noted that routine folic acid supplementation can mask B12 deficiency, delaying diagnosis and treatment.(Ononogbu et al. 2024) A systematic review found that most individuals with SCD on regular folic acid had normal or increased plasma folate, but no discernible impact on haemoglobin, infections, or pain crises; evidence on benefits is insufficient, and large trials are needed.(Arrey Agbor et al. 2024) Folate metabolism may play a role beyond simple haematological support, although the evidence remains preliminary.(Nwofor et al. 2025) A 2025 prospective single-arm study of 60 children with SCD (mean age 9.2 years) found that all patients were zinc-deficient at baseline (mean zinc level 42.8 ± 14.1 µg/dL). After 12 months of daily oral zinc sulphate (10–30 mg depending on age), weight and height Z-scores improved significantly (*p* < 0.001), and the frequencies of vaso-occlusive crises, infections, hospitalisations, and transfusions reduced significantly (*p* < 0.001).(Youssry et al. 2025) Plasma zinc and magnesium levels were significantly lower in individuals with SCD than in controls.(Yousef et al. 2025) A narrative review highlighted that zinc monitoring is essential for reducing morbidity and infections in children and adolescents with SCD but noted limited data for adults and called for new adult research.(Miranda et al. 2022) A 2025 study protocol describes a randomised, placebo-controlled trial of zinc for infection prevention in Ugandan children, addressing a research gap in African paediatric populations.(Namazzi et al. 2023) Zinc may increase haemoglobin level (MD 1.26 g/dL, 95% CI 0.44 1.26; low certainty evidence) but may not reduce the frequency of crisis.(Bolarinwa et al. 2024) Overall, observational studies have reported low zinc levels and associations with adverse clinical outcomes, while small intervention studies suggest potential benefits of supplementation. However, limited sample sizes and scarce evidence of adult-specific interventions preclude firm conclusions regarding routine supplementation. Studies of n-3 supplementation have reported favourable effects on selected inflammatory and clinical outcomes in SCD.(da Conceicao Souza et al. 2025) A 2025 case-control study also reported favourable findings among patients receiving omega-3 supplementation alongside conventional treatment.(Obied et al. 2025) Although individual randomised trials have reported favourable effects of omega-3 supplementation on selected outcomes, the evidence from systematic reviews remains of very low certainty for most assessed outcomes. A 2025 double-blind RCT of 40 children with SCD and vaso-occlusive pain episodes found that L-arginine (100 mg/kg three times daily) significantly decreased pain score at day 3 of admission (*p* = 0.036), total analgesic use (*p* < 0.001), time to crisis resolution (*p* = 0.011), length of hospital stay (*p* = 0.016), and tricuspid regurgitant jet velocity (*p* < 0.001).(Abd Elglil et al. 2025) L-arginine significantly improved pain intensity, vaso-occlusive crises and inflammation (*p* < 0.05).(Orsi et al. 2025) The Cochrane review found that L-arginine may reduce pain severity (MD -1.41, 95% CI -1.65 to -1.18; low-certainty) but may not reduce crisis frequency.(Bolarinwa et al. 2024) Vitamin C (1400 mg) plus vitamin E (800 mg) versus placebo (very low certainty) may increase the risk of acute chest syndrome (RR 2.66) and may not reduce the frequency of crises (RR 1.18) or pain severity (RR 1.33). For N-acetylcysteine (NAC) 1200 mg, the review found that NAC may not reduce crisis frequency (rate ratio 0.99, 95% CI 0.53 to 1.84), pain severity (MD 0.17, 95% CI -0.53 to 0.87), or haemoglobin level (MD -0.18 g/dL, 95% CI -0.40 to 0.04), with all evidence rated low or very low certainty.(Bolarinwa et al. 2024) Overall, experimental evidence suggests a potential benefit of L-arginine for pain-related outcomes; however, the evidence remains limited and of low certainty for some outcomes.

### Age-related differences and research gaps

A notable finding of this review is the disparity in the availability of evidence between children, adolescents, and adults. For vitamin D, the meta-analysis reported a pooled prevalence of deficiency of 56.2% in children and 75.5% in adults (*p* < 0.001). (Mohamed et al. 2020) For zinc, the lack of adult data was explicitly highlighted; this gap is now partially addressed by newer adult studies.(Miranda et al. 2022) The 2024 cross-sectional study on B12 deficiency in adults (*n* = 100) found a prevalence of 27%, providing one of the few adult-specific estimates for this nutrient.(Ononogbu et al. 2024) The large Tanzanian cohort study on vitamin B12 included adults but did not provide separate prevalence estimates by age group, although severely low levels were observed across all age groups.(Kisali et al. 2024) The prospective longitudinal study focused exclusively on adults (*n* = 62) and found that 70% of patients had deficiencies in vitamin D, B12, and zinc, and that protein-energy and micronutrient deficiencies were independent predictors of severe SCD, increased hospitalisations, and reduced health-related quality of life.(Kamal et al. 2021).

### Nutritional interventions and clinical outcomes

Several studies have evaluated nutritional interventions in SCD, with findings varying across interventions and outcomes. A systematic review reported potential benefits of fatty acids, L-arginine and vitamin D3 for selected clinical outcomes.(Orsi et al. 2025) while a qualitative systematic review reported favourable outcomes with DHA and EPA supplementation.(da Conceicao Souza et al. 2025) A 2025 trial of L-arginine in children with VOE reported significant reductions in pain score, analgesic use, crisis-resolution time, hospital stay, and tricuspid regurgitant jet velocity ( TRJV).(Abd Elglil et al. 2025) A 2025 zinc supplementation trial in children reported improvements in growth and selected clinical, haematological, and oxidative parameters.(Youssry et al. 2025) A case-control study also evaluated omega-3 fatty acids as an adjuvant intervention in SCD.(Obied et al. 2025) A chapter on nutritional therapy concluded that it is a vital component in managing inherited blood disorders and should be integrated into standard care as part of a multidisciplinary approach.(Karimi 2025) A scoping review on traditional medicine in SCD identified several medicinal plants with anti-sickling, antioxidant, anti-inflammatory, and immunomodulatory properties, and some formulations showed potential to reduce vaso-occlusive crises and improve haematological parameters.(Javed et al. 2026) A study on food deserts and SCD highlighted the need to address food accessibility as a social determinant of nutritional status in SCD.(Scott and Cohen-Young 2025) The evidence base spans more than two decades, and several earlier studies involved relatively small samples. Older studies were retained where contemporary evidence was limited, but their findings were interpreted alongside more recent evidence and with consideration of study design, sample size, methodological quality, and current applicability. Publication date alone was not considered an indicator of evidence quality; where available, greater interpretive weight was given to methodologically stronger and higher levels of evidence.

## Strengths and limitations

This review has several strengths, including comprehensive mapping of evidence across systematic reviews, meta-analyses, randomised controlled trials, cohort studies, and cross-sectional studies, as well as age-stratified analyses (children, adolescents, adults) that reveal important differences in deficiency prevalence and evidence availability. However, several important limitations must be acknowledged. The overall evidence base is characterised by small sample sizes and predominantly descriptive study designs, with a stark dearth of interventional studies. Sex-specific differences were not consistently examined across the included studies, limiting assessment of whether nutritional requirements and deficiency patterns differ between males and females. Substantial heterogeneity in study designs, outcome definitions, supplementation regimens, dosages and durations limits comparability and precludes definitive meta-analyses for many nutrients. The certainty of evidence for most nutritional interventions was rated as low or very low, primarily due to risk of bias, imprecision and indirectness. Adult-specific data are notably scarce for several critical nutrients, particularly zinc. Geographic disparities exist, with most research concentrated in West Africa (particularly Nigeria) and North America, while large SCD-burdened regions in East, Central and Southern Africa, as well as in India, remain understudied. Many studies assessed vitamin D status at a single time point without accounting for seasonal variation, potentially leading to misclassification.

## Conclusion

The available evidence indicates increased energy and protein requirements in SCD, while adequate carbohydrate intake remains important for meeting overall energy needs. Frequently reported micronutrient deficiencies include vitamin D, zinc, vitamin B12, folate, and vitamin C. Nutritional interventions, including omega-3 fatty acids, L-arginine, zinc, and vitamin D, have shown potential benefits for selected outcomes; however, the overall evidence remains limited and is frequently of low or very low certainty. Further well-designed, adequately powered trials are needed before definitive SCD-specific nutritional and supplementation recommendations can be established.

## Recommendations

Nutritional assessment should be integrated into comprehensive SCD care, with interventions individualised according to age, nutritional status, laboratory findings, and clinical condition. Existing nutrition programmes may provide platforms for nutritional support; however, their effectiveness in SCD-specific interventions remains unestablished. Further high-quality trials are needed to define age-specific nutritional requirements and evaluate the efficacy and safety of targeted supplementation before population-level recommendations can be made. 

## Data Availability

No datasets were generated or analysed during the current study.
